# Supplementary material for: Finetuning Foundation Models for Temporal Clinical Transcriptomics Data
Source: Bioinformatics. 2026 Aug 27;42(9):btag640. doi: 10.1093/bioinformatics/btag640 (PMC13576074; doi:10.1093/bioinformatics/btag640)
Supplement: Supplementary file 1 [file btag640_supplementary_data.pdf]

Finetuning Foundation Models for Temporal Clinical Transcriptomics Data

Supplementary Information

1 Introduction

Timeseries clinical transcriptomic datasets allow understanding of the dynamics of disease progression/treatment response in patients and help to identify drug targets and discover disease endotypes. In most clinical trials or observational studies, patients show variable response to drug treatment [ref]. While some patients benefit from the drug (responder group), others show limited to no response (non-responder group). This could be due to various reasons such as differences in genetic characteristics, presence of distinct disease subtypes, environmental factors or differences in disease severity. It is important to understand the reasons for response/non-response at the molecular level so that new drug targets can be found to benefit patients who do not respond to standard therapies, which is very challenging. In addition to the high resolution of the expression data and the small number of individuals that are profiled, which often makes it hard to correctly identify an underlying cause, clinical transcriptomic datasets tend to be noisy because of heterogeneity in patient demographics, disease state, environmental factors, etc.

In this study, we leverage gene embeddings from a text-based foundation model (GenePT (Chen and Zou 2025)). We finetune GenePT with static, general tissue-specific gene expression to capture the context of a specific tissue. We then use the context-specific embeddings as part of a GNN to model transcriptomic timeseries datasets. We use GNNs for finetuning the embeddings and for temporal modeling using protein-protein interaction network as the edges in the graph. We applied our method to three clinical time series datasets representing interventions to treat different diseases. As we show, our method accurately recovers known genes/biological mechanisms that underlie disease progression and response and also raises novel hypothesis about mechanisms associated with non-response to treatment.

2 Datasets

We used 3 diseases ulcerative colitis (UC), Crohn's disease (CD), and psoriasis to test our method. We did not use any of the healthy control samples and instead relied on

the pretreatment samples as baselines for all 3 diseases. Following is the procedure followed for selection of samples.

2.0.1 GSE171012 (Psoriasis)

The metadata was extracted, and the following filtering criteria were applied in sample selection. - Column used.for.analysis.ch1 had a value "used for analysis: TRUE" - Column cell.type.ch1 = "bulk" - Column characteristics_ch1.4 = "tissue: lesion" - Column clinical status:ch1 = "Psoriasis_PreTreatment" or "Psoriasis_SecukinumabTreatmentWeek4" or "Psoriasis_SecukinumabTreatmentWeek12" The batch information given in column "sequencing batch:ch1" of the metadata was used for correcting the batch effect. One patient "PSO38 was removed as it was found to be an outlier. 36 samples were obtained for patients who underwent treatment.

2.0.2 GSE171770 (Ulcertive Colitis and Crohn's Disease)

The clinical information file from NCBI-GEO was used to select samples belonging to Ulcerative Colitis (UC) and Crohn's disease. The Mayo scores for UC and CDAI scores for Crohn's were also selected. After selecting the patients, gene expression for the patients for mucosal intestine tissue were extracted from the series file. There were 9 UC and Crohn's disease patients.

Patients who completed all visits were chosen. The final number of patients in each study are reported in Table 1 in the main paper.

2.1 Healthy Tissue Samples

Healthy tissue samples were extracted from NCBI-GEO. These samples were used for finetuning the foundational gene embeddings from GenePT. Table-S1 lists the datasets and the number of healthy samples that were extracted. A total of 183 samples for colon and 77 samples from skin were collected.

Table 1 List of Studies for Healthy Samples

Tissue	NCBI GSE IDs	#Samples
Colon	GSE100179, GSE112366, GSE166427, GSE66407, GSE87473	20, 26, 50, 66, 21
Skin	GSE130633, GSE178197, GSE203415, GSE284467	30, 15, 10, 22

We made the decision not to combine microarray and sequence count data as there could be normalization issues. Samples for colon came from microarrays (Affymetrix, Illumina, Agilent). The normalized expression values were extracted using the GEOQuery library (Davis and Meltzer 2007). Annotation files associated with the dataset were extracted and all datasets were harmonized to have gene symbols. In case of multiple transcripts for a given gene symbol, transcript with maximum expression value was extracted. The samples for skin consisted of counts from sequencing datasets predominantly generated by 10x genomics. Counts were extracted and gene identifiers

were mapped to the gene symbols. In case of multiple measurements for a given gene symbol, the maximum count value was retained. TPM normalization (Dillies et al. 2013) was applied to the count data. Samples collected from different datasets for a tissue were assigned different batch numbers and PyComBat (Behdenna et al. 2023) was used for batch correction.

3 Tissue Enrichment Comparison

The finetuned embeddings were extracted from the model for each tissue (skin and colon) from the GAT model. Since it is a 3-layered model, embeddings of 756, 256, and 128 dimensions were obtained. PCA was applied to all 3 embeddings and 50 components were extracted. This was fed into a umap to obtain a 2-d visualization and K-Means (k=10) was applied. The clusterings of the 3 embeddings were then fed into the Tissue Enrichment tool (Jain and Tuteja 2018) to find if any of the embeddings were enriched in the tissue of interest. Depending of which embedding showed tissue enrichment, it was selected for initialization of the temporal model. Figure S1 shows the tissue enrichment of 1536-dimensional GenePT and 756-d embeddings of skin. Since K-Means gave 10 clusters, the largest cluster was chosen to find the tissue enrichment.

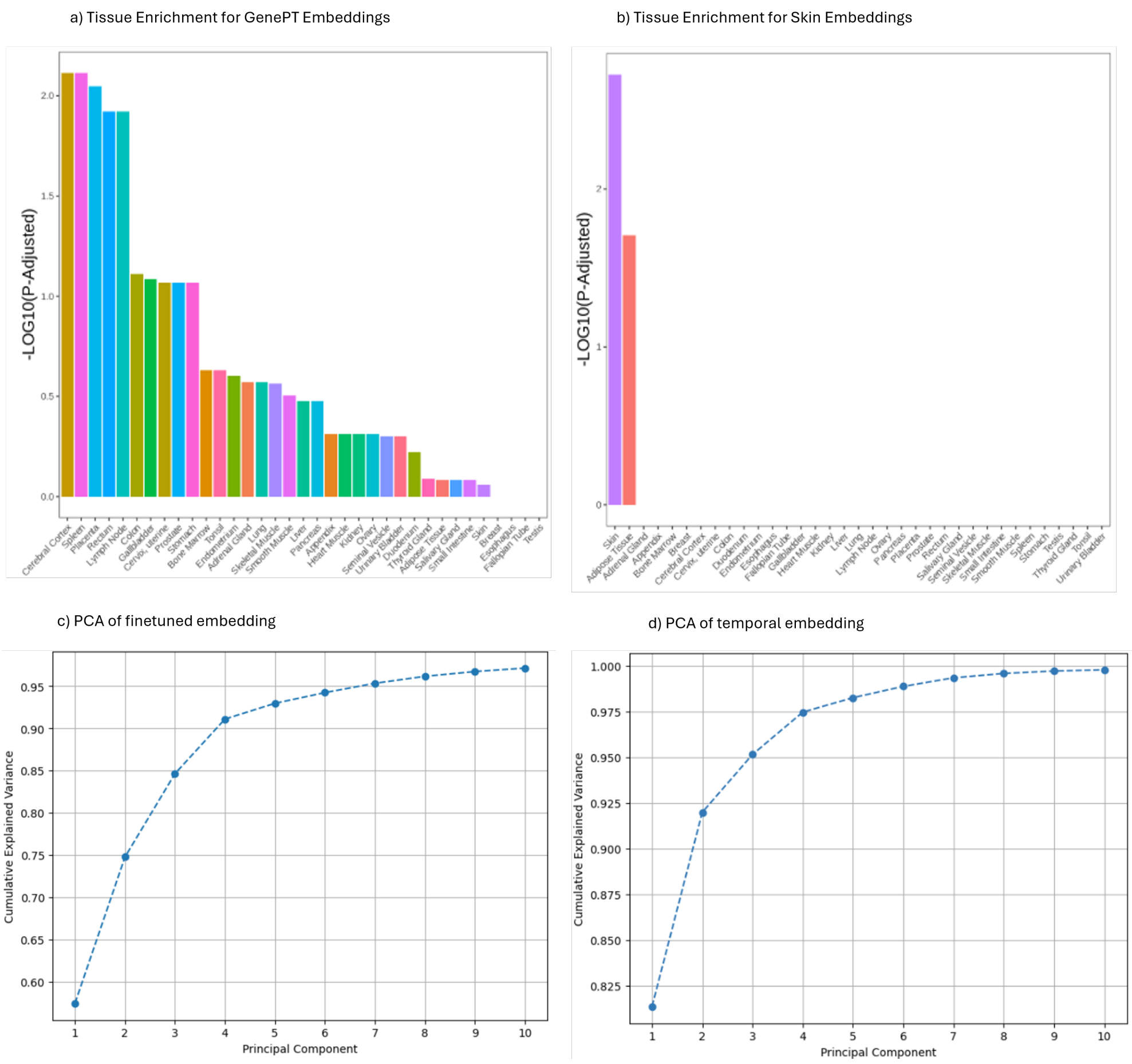

Fig. 1 Tissue Enrichment of embeddings. a) Tissues enriched in 1536 dimensional GenePT embeddings. b) List of tissues enriched in the 756 dimensional embedding of skin obtained after finetuning of the original GenePT embeddings. c) PCA of the 756-d finetuned embedding for skin. d) PCA of the 16-d temporal embedding for psoriasis responders at pretreatment.

4 Results of Crohn's Disease

Figure-S2 shows the top 5 GO-Processes enriched in responders and non-responders in Crohn's Disease. If the GO-processes were similar, then only the most specific ones were shown. Although there are immune related GO-processes that are up regulated in responders, there is also up regulation in healing activity - endodermal cell differentiation (GO:0035987), and positive regulation of lipid kinase activity (GO:0090218). The non-responder group mainly show up regulation in immune activity confirming lack of effect of IL-6 inhibitor.

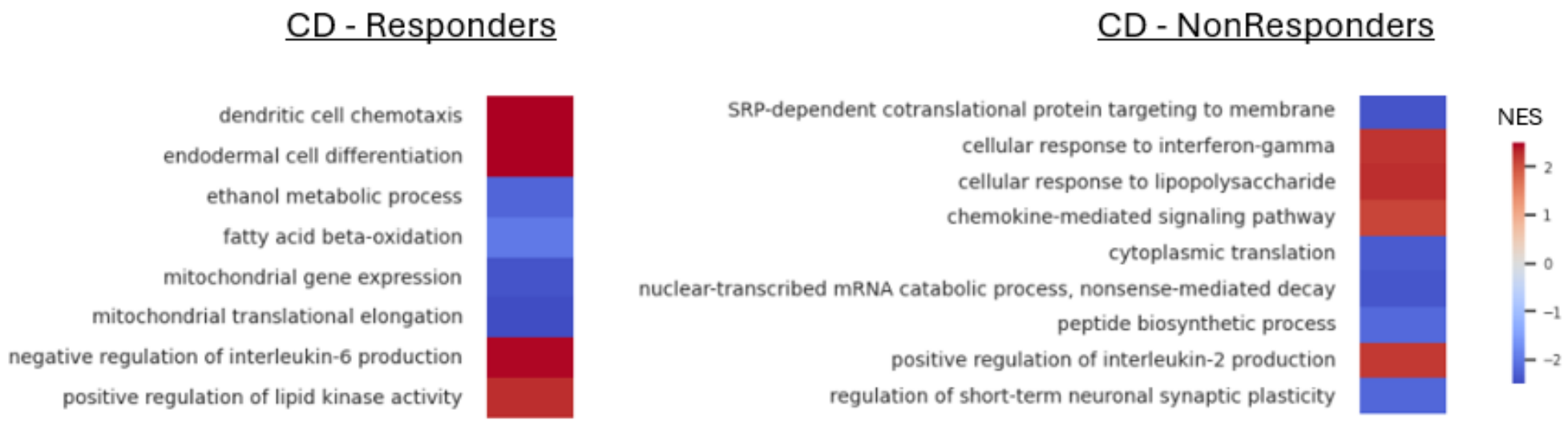

Fig. 2 GSEA results for Crohn's Disease. Top GO-Process results for responder and non-responder groups. FDR < 0.05, NES: Normalized Enrichment Score.

5 Results of Alopecia Areata

Table-2 (at the end of the references) lists the results of responders in the treatment of alopecia areata.

6 Percentage of Masked Genes for Finetuning

The effect of the proportion of masked genes on finetuning is shown in Figure-3.

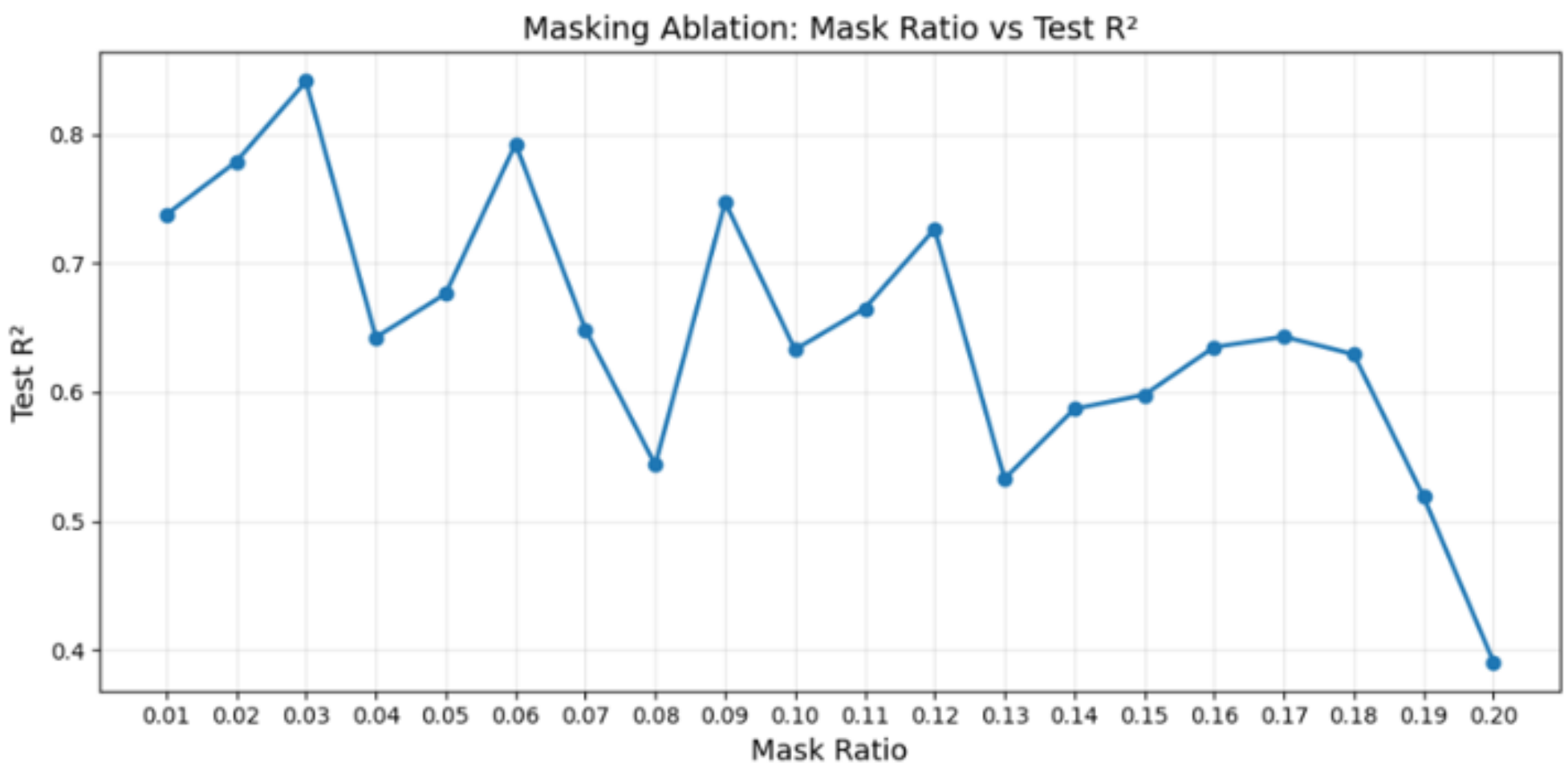

Fig. 3 Shows the % of masked genes Vs the R-squared of the model on the test set.

7 Comparison Between PPI Networks

The String and IntAct PPI networks are compared across 4 datasets for the detection of remission-related genes and primary/partial mechanism of treatment. Results are listed in Table-3 (at the end of the references)

8 Results - Gene Lists

Here are the results of differential expression (FDR < 0.05) across the 3 studies. The gene symbol is listed along with the log2 fold change within parenthesis. The fold change was calculated by comparing the gene expression at the final timepoint and pretreatment, with the later as the baseline across all patients.

UC: CA3(0.726), CCL20(0.671),CD19(0.168), CD2(0.428), CXCL1(-2.877), CXCL10(-1.836), CXCL13(-0.686), CXCL9(-1.533), CXCR2(-4.528), CXCR4(0.418), DEFA5(1.476), FAM151A(3.586), IL1B(-2.573), LCK(-0.084), REG1A(-2.900), REG1B(-4.980), REG3A(-2.204), THSD7B(-0.461), TIMP2(0.070), ZAP70(-0.102).

CD: More than 300 genes were detected to be differentially expressed. Genes below have FDR < 0.001.

ABO(-2.66), ACVR2A(-0.84), ADAMTS8(-0.74), ADAMTSL3(-0.57), ADH1C(-3.97), ADH4(-2.37), ADM5(-0.54), AMER3(-3.16), ANKLE1(1.25), ANO9(-1.66), APBB1(1.51), ATP10A(1.09), ATP10D(0.53), BGN(2.35), BHLHE22(2.91), BRICD5(-0.83), C1QA(1.0), CAPN14(0.35), CCRL2(-0.49), CD2(1.57), CD247(1.52), CDK15(1.35), CEP135(0.71), CLC(0.98), CLCNKB(-1.4), CLDN1(-0.92), CLDN4(-2.47), CLRN3(-2.77), COL11A1(1.65), COL12A1(3.57), COL16A1(0.76), COL1A1(2.31), COL27A1(1.9), COL3A1(2.54), COL4A2(1.85), COL5A1(1.92), COL5A2(2.34), COL6A1(1.7), COL6A3(2.19), COLGALT1(0.65), COLGALT2(0.39), CYP2E1(0.71), DCST2(-1.21), DCSTAMP(1.59), DDX39A(0.07), DEFA4(0.01), DEFA6(-3.3), DLEU7(2.08), DPY19L2(1.47), DUOXA1(-2.54), DUOXA2(-1.95), ELN(0.19), ELOVL3(2.06), ENDOU(0.01), EQTN(-0.42), EVI2B(2.31), EXTL3(0.54), FADS6(-1.64), FAM151A(-1.5), FAM53A(1.11), FBN1(2.12), FBXO39(0.43), FER1L5(-1.54), FOXE3(0.43), GALNT15(2.9), GARNL3(-0.06), GGN(2.42), GPR137C(-0.3), GPR31(-0.68), GREB1L(-1.84), HRH4(2.24), HS3ST6(-0.76), HSPB7(0.3), ICAM1(1.4), IGFN1(-1.08), IL2(2.23), ISM1(2.97), ITGA4(1.22), ITGA8(0.2), ITPA(-0.22), KLLN(-0.04), LAMC1(1.22), LAMC3(2.33), LGALS3(-1.42), LGALS8(0.22), LIPE(-0.37), LMOD1(-0.34), LTBP1(0.92), LTBP2(2.27), LTF(1.91), LUM(2.17), LYG1(0.45), MEGF11(3.5), MEP1B(-1.45), MFAP5(-1.65), MIOX(2.28), MKRN2(0.15), MLN(-1.72), MMP2(2.59), MMP21(2.05), MMP9(3.43), MOGAT3(-2.92), MPP4(2.05), MS4A2(0.84), MS4A8(-3.51), NAB1(0.36), NDST3(0.32), NOX5(-1.2), PDZD2(0.82), PKHD1L1(0.94), PLA2G12A(-0.59), PLA2G4D(-1.28), PLA2G5(1.76), PLA2R1(0.72), PLAUR(0.78), PLCXD3(-1.92), PLOD1(0.59), PLXNB3(0.06), PNCK(-3.11), POSTN(1.15), PRCD(0.67), PRR19(0.6), PTCH1(-0.62), PTGDR2(0.03), RIPPLY3(-0.23), RNF175(2.5), RNF182(3.23), RNFT2(1.03), RPS27L(-0.24), S1PR1(2.15), SEMA4A(1.46), SEMA5A(-0.25), SERPINB8(0.07), SH3TC2(-0.32), SHH(-1.73), SLC22A1(0.62), SLC35D3(-0.23), SLC4A11(0.5), SLC52A1(-1.64), SLC5A11(-0.22), SLC5A7(-0.46), SLC5A9(-1.2), SPATA22(0.07), ST8SIA4(1.77), SVOP(-3.3), SYK(0.39), THSD7B(0.8), TJP3(-2.51), TLR2(2.06), TM6SF1(1.8), TMEFF2(-0.97), TMEM117(-0.13), TMEM132B(0.15), TMEM163(2.63), TMEM178A(-0.74), TMEM198(-0.52), TPSD1(0.15), TRAM1L1(1.4), TSPAN5(1.3), TVP23A(1.32), TYROBP(1.41), VCAN(1.29), VSTM2L(-0.07), XKR6(-0.31), YTHDC1(-0.07), ZAP70(1.3), ZNF311(-1.02), ZNF385B(2.03), ZNF391(0.46), ZNF491(-0.1), ZNF575(-2.22), ZNF732(-1.95), ZNF804A(1.84), ZNF883(0.81)

Psoriasis: More than 800 genes were detected to be differentially expressed. Listing a subset of genes with FDR < 0.001.

ARID4A(0.654), ASPM(-0.658), AURKA(-1.252), AURKB(-1.635), BCAN(2.088), BGN(1.176), BIRC5(-1.574), BRCA1(-0.234), BUB1(-0.999), BUB1B(-1.045), CCNA2(-1.395), CCNB1(-1.886), CCNB2(-1.439), CCNE1(-1.782), CCR1(0.014), CD247(-0.299), CD274(-2.233), CD3E(-0.316), CDC20(-1.808), CDC45(-1.826), CDC6(-1.457), CDK1(-1.189), CDT1(-0.862), CENPA(-0.889), CENPE(-1.054), CENPF(-0.965), CFH(1.871), CHEK1(-0.426), COL12A1(1.389), COL14A1(1.04), COL16A1(1.048), COL5A2(1.004), CREBBP(0.616), CRTAP(1.034), CXCR2(-1.895), DLGAP5(-1.45), EXO1(-1.115), FBLN2(1.432), FBLN5(1.493), FBN1(1.391), FGFR1(1.547), FMOD(0.646), FOXM1(-1.499), FYN(1.222), GAN(2.271), HJURP(-1.286), HLA-DRA(-0.071), HLA-F(-0.053), HNRNPL(0.08), HSPG2(0.979), IFI44(-1.855), IFI44L(-1.452), IFIH1(-1.014), IFIT1(-1.264), IFIT3(-1.467), IL1B(-2.604), IRF1(-0.503), IRF7(-2.017), ISLR(1.636), ITGA1(0.915), ITGA11(1.79), KIF11(-0.589), KIF14(-0.297), KIF20A(-1.781), KIF23(-0.983), KIF2C(-1.581), KLK10(-2.802), KRAS(0.395), KRT25(2.987), KRT27(2.622), KRT31(2.035), KRT34(0.354), KRT71(2.924), KRT75(0.214), KRT77(2.821), KRT79(1.583), KRT81(1.714), LAMA5(0.892), LAMB2(1.089), LAMC1(1.028), LCK(-0.405), LCN2(-5.192), LPAR1(1.438), LRP5(0.581), LRP6(0.897), LTBP1(0.57), MAD2L1(-0.846), MAVS(0.054), MCM10(-1.4), MCM3(-0.239), MCM6(-0.307), MCM7(-0.558), MMP9(-1.575), MVK(-0.198), MX1(-2.522), MYCN(0.047), NEK2(-1.126), NID1(0.794), NOD2(-1.225), NR3C1(1.074), NRAS(0.704), OAS3(-1.819), OASL(-3.953), PARP9(-1.24), PDGFB(1.061), PDGFRA(1.421), PLK1(-1.977), PLS1(0.049), PNP(-1.435), POLD1(-0.287), POLE2(-0.355), PPARG(1.19), PPP1CB(0.591), PTEN(0.576), PTPRK(0.611), RAD51(-0.931), RND1(-2.745), RPA3(-0.032), RPS10(-0.371), RRM2(-2.154), S100A7(-5.147), SCGB2A2(2.573), SERPINA12(3.185), SERPINB4(-8.003), SLC37A4(-0.047), SPDL1(0.046), TGFB1(0.127), TGFB3(1.164), TGFBR3(1.642), TLN2(1.176), TNF(-0.161), TPX2(-0.871), TRIP13(-1.095), UBE2T(-0.977), VCAN(1.083), XPO1(0.465), ZWINT(-1.341).

References

Behdenna, A., Colange, M., Haziza, J., Gema, A., Appé, G., Azencott, C.-A., Nordor, A.: pycombat, a python tool for batch effects correction in high-throughput molecular data using empirical bayes methods. BMC bioinformatics **24**(1), 459 (2023)

Chen, Y., Zou, J.: Simple and effective embedding model for single-cell biology built from chatgpt. Nature biomedical engineering **9**(4), 483–493 (2025)

Davis, S., Meltzer, P.S.: Geoquery: a bridge between the gene expression omnibus (geo) and bioconductor. Bioinformatics **23**(14), 1846–1847 (2007)

Dillies, M.-A., Rau, A., Aubert, J., Hennequet-Antier, C., Jeanmougin, M., Servant, N., Keime, C., Marot, G., Castel, D., Estelle, J., *et al.*: A comprehensive evaluation of

normalization methods for illumina high-throughput rna sequencing data analysis. Briefings in bioinformatics **14**(6), 671–683 (2013)

Jain, A., Tuteja, G.: TissueEnrich: A tool to calculate tissue-specific gene enrichment (2018)

Table 2 Results for Alopecia Areata

Term	fdr_WK4	nes_Wk4	fdr_Wk12	nes_Wk12
Positive Regulation Of Cytokine Production (GO:0001819)	0.10	-1.65	1.00	1.00
Cytoplasmic Translation (GO:0002181)	0.74	-1.23	0.02	-1.64
Antigen Processing And Presentation Of Exogenous Peptide Antigen (GO:0002478)	0.02	-1.72	1.00	-0.74
rRNA Processing (GO:0006364)	1.00	1.17	0.00	-1.88
Translation (GO:0006412)	0.73	-1.23	0.01	-1.67
Macromolecule Biosynthetic Process (GO:0009059)	0.73	-1.23	0.01	-1.66
Gene Expression (GO:0010467)	0.83	-1.13	0.00	-1.73
Immunoglobulin Mediated Immune Response (GO:0016064)	0.01	-1.74	1.00	1.12
rRNA Metabolic Process (GO:0016072)	1.00	1.28	0.00	-1.86
Ribonucleoprotein Complex Biogenesis (GO:0022613)	0.95	-0.98	0.01	-1.70
Myeloid Cell Differentiation (GO:0030099)	0.02	1.00	0.47	1.60
Maturation Of SSU-rRNA (GO:0030490)	0.13	-1.62	0.01	-1.71
Melanosome Organization (GO:0032438)	0.08	0.79	0.02	1.65
Positive Regulation Of T Cell Proliferation (GO:0042102)	0.02	-1.70	1.00	0.94
T Cell Activation (GO:0042110)	0.07	-1.66	1.00	1.21
Ribosome Biogenesis (GO:0042254)	0.88	-1.10	0.01	-1.70
Ribosomal Small Subunit Biogenesis (GO:0042274)	0.30	-1.46	0.03	-1.63
Regulation Of Tyrosine Phosphorylation Of STAT Protein (GO:0042509)	0.03	-1.69	1.00	1.31
Positive Regulation Of Tyrosine Phosphorylation Of STAT Protein (GO:0042531)	0.05	-1.67	0.95	1.50
Peptide Biosynthetic Process (GO:0043043)	0.74	-1.23	0.01	-1.68
Cellular Respiration (GO:0045333)	0.08	-1.64	1.00	-0.94
Positive Regulation Of Immune Response (GO:0050778)	0.01	-1.75	1.00	1.29
Antigen Receptor-Mediated Signaling Pathway (GO:0050851)	0.00	-1.78	1.00	1.36
T Cell Receptor Signaling Pathway (GO:0050852)	0.00	-1.77	1.00	1.40
Positive Regulation Of T Cell Activation (GO:0050870)	0.01	-1.75	1.00	0.94
Regulation Of Neutrophil Chemotaxis (GO:0090022)	0.09	-1.65	1.00	-0.93
Regulation Of Signal Transduction By P53 Class Mediator (GO:1901796)	1.00	1.30	0.05	-1.61
Negative Regulation Of Proteolysis Involved In Protein Catabolic Process (GO:1903051)	1.00	1.29	0.04	-1.61

Table 3 Results Comparison Between String and Intact PPI Networks

Disease	Detection	String	IntAct
Ulcerative Colitis	# Genes (Enchr)[1]	15 (1e-9)	23 (0.001)
	Treatment mechanisms[2]	yes	yes
Crohn's Disease	# Genes (Enchr)	11 (0.009)	11 (0.024)
	Treatment mechanisms	yes	partial
Psoriasis	# Genes (Enchr)	40 (1e-20)	9 (0.017)
	Treatment mechanisms	partial	partial
Alopecia Areata	# Genes (Enchr)	31 (2e-15)	19 (3e-5)
	Treatment mechanisms	partial	partial

[1] # Genes: Number of remission-related genes detected at pvalue < 0.05, (Enchr): p-value of enrichment of remission-related genes

[2] Treatment mechanisms: Detection of biological processes related to the direct treatment mechanism of action. ND: Not detected
